# Free amino acid content in breast milk of adolescent and adult mothers in Ecuador

**DOI:** 10.1186/2193-1801-3-104

**Published:** 2014-02-21

**Authors:** Manuel E Baldeón, Julie A Mennella, Nancy Flores, Marco Fornasini, Ana San Gabriel

**Affiliations:** Facultad de Ciencias de la Salud, Universidad de las Américas, Quito, Ecuador; Monell Chemical Senses Center, 3500 Market Street, Philadelphia, PA 19104-3308 USA; Colegio de Agricultura Alimentos y Nutrición, Universidad San Francisco de Quito, Quito, Ecuador; Ajinomoto Co., Inc, Tokyo, Japan

**Keywords:** Human milk, Free amino acids, Glutamine, Glutamate, Lactation

## Abstract

Because of increased incidence of teenage births and high prevalence of lactation in Latin America, we determined the patterning of free amino acids (FAAs) in breast milk of 65 primiparous Ecuadorian women of varying ages (14–27 years). An automatic amino acid analyzer quantified levels of FAAs in milk samples obtained at three lactation stages: colostrum, transition, and mature milk. Regardless of mother’s age, most FAAs increased with time postpartum, with taurine, glutamic acid, glutamine, and alanine being most abundant in all stages.

## Introduction

Exclusive breastfeeding for the first six months of life optimizes infant growth, development, and health and is beneficial to maternal health. Breast milk contains adequate nutrients, minerals, vitamins, and water for growing, but for reasons not completely understood, the mammary tissue of most mammals produces large amounts of nonprotein nitrogenous compounds, including free (protein-unbound) amino acids (FAAs) (Armstrong and Yates 1963; Svanberg et al. 1977; Lei et al. 2012).

Albeit limited, most research to date quantifying sources of nonprotein nitrogen in breast milk has focused on adult mothers. During recent years, teenage pregnancy has become a rising public health concern in many countries (Moran 2007), having significant social and reproductive consequences (Chedraui 2008). For Latin American countries such as Ecuador, adolescent pregnancies now account for almost one fifth of all pregnancies (UNFPA 2010). Because the vast majority of these women initiate breastfeeding (Ordóñez et al. 2005), the present study quantified concentrations of FAAs in milk collected at three stages of lactation and tested the hypotheses that the time-related changes in the patterning of FAA profiles would differ as a function of maternal age.

## Materials and methods

During 2009–2011, we recruited 65 non smoking women who gave birth at Hospital Gíneco-Obstétrico Isidro Ayora of Quito, a public obstetric hospital in Ecuador that serves low-income patients. Inclusion criteria included good health for dyad, singleton term birth without complications, and mother’s intent to reside locally and exclusively breastfeed for at least 4 months. The study was approved by the Human Subjects Protection Committee at the Universidad San Francisco de Quito, and informed consent was obtained from each participant before participation. At study entry, all but 10 of the babies were weighed and all but 3 women were weighed and measured while wearing light clothing and without shoes.

Milk samples were collected after an overnight fast and during 06:00–08:00 hours at three time points: colostrum (3.3 ± 0.2 days postpartum; range = 0–7), transition milk (15.1 ± 0.1 days postpartum; range = 13–17), and mature milk (2 and/or 4 months postpartum). Colostrum was collected at the delivery hospital (26%) or subjects’ homes (74%); transitional and mature milk samples were collected at subjects’ homes.

Mothers used a breast pump to collect a minimum of 10 mL milk from each breast, which was pooled into a 50-mL Falcon aseptic tube. Samples were transported at 4°C to the laboratory within 2 h, and proteins were immediately (within 2 h) precipitated with 6% 5-sulfosalicylic acid dihydrate and stored in liquid nitrogen until assayed for amino acids (Noguchi et al. 2006). Samples were sent to Japan in dry ice; each sample arrived frozen and was stored at −70°C until analysis. The supernatant was purified with a 0.45-μm filter; molecules >10 kDa were removed with an Amicon Ultra centrifugal filter (Millipore, Tokyo, Japan) and injected into an automatic amino acid analyzer (model L-8900; Hitachi, Tokyo, Japan) for cation-exchange chromatography separation. Each amino acid was detected by spectrophotometric analysis with ninhydrin reagent (Noguchi et al. 2006). The system has a high-reproducibility retention time with coefficient of variation (CV) 0.3% (arginine), peak area with CV 1.0% (glycine, histamine), and limit of detection (LOD) of 3 pmol [signal-to-noise ratio (S:N) = 2; asparagine]. All samples were measured in duplicate; reproducibility was verified by measurements of standard preparations. Amino acid standards were intercalated with each sample to ensure reproducibility ≥98% or CV <2%. LOD varied from 2–10 μmol/L and was specific to each amino acid. Values <3-fold the S:N were considered to be in the LOD.

### Statistical analyses

A mixed effects model including fixed effects for time point (stage of lactation: colostrum, transition, mature), maternal age (as a continuous variable), and random subject effect with a compound symmetry covariance structure were created to examine the relationship between stage of lactation, FAAs and maternal age. For each individual, the *last* observed value for that particular FAA (month 2 or 4) was used as value for mature milk. Least squares means (LSM: model-adjusted means) were calculated from mixed effects model with time point (stage of lactation) and other covariates as fixed effect and subject as the random effect. The differences in LSM between all time points were compared using a *t*-test. The models in this analysis were developed for each individual FAAs, as well as grouping of FAAs into Indispensible (Essential) or Dispensible (Non-Essential) or Total based on the classifications of the Dietary Reference Intakes for Women (Otten et al. 2006). Statistical significance was defined as P < 0.05. All data are reported as LSM (± standard error).

## Results

The 65 mothers were 14–27 years old (mean: 18.4 ± 0.4; 60% ≤ 18), with average body mass index of 24.6 ± 0.4 kg/m^2^ (normal weight, 58.1%; overweight, 35.5%; obese, 6.5%). Twenty-eight of the 65 infants (43.1%) were female. For the 55 babies with weight data, weight-for-age z-scores averaged −0.29 ± 0.17 (range: -2.76 to 4.57; more than half (62%) had z-scores <0.

We obtained a colostrum sample from each woman, transitional milk from 48, and mature milk from 45. There was no significant association between age and missing values for any time point. FAA levels changed significantly with increasing lactation (Table 1). Most FAAs (histidine, isoleucine, leucine, methionine, phenylalanine, threonine, valine, cysteine, tyrosine, alanine, aspartic acid, glutamic acid, glutamine, glycine, serine) increased as lactation progressed, four decreased (lysine, arginine, taurine, proline) and two were undetectable at any time point (tryptophan, asparagine). Glutamic acid, glutamine, taurine and alanine were most abundant in milk at each of the three stages of lactation. Both nonessential and essential FAAs increased as lactation progressed. Maternal age did not have a significant linear relationship with any of the FAAs (Table 1).

**Table 1:** **Free amino acid content (μmol/dL)** ^**1**^ **of human milk collected during three stages of lactation**

	Stage of lactation:			
	Colostrum	Transition	Mature	Statistical analyses: effect of maternal age (p value)
**Essential FAAs** ^**2**^ **:**				
Histidine	1.45 ± 0.15^a,3^	2.31 ± 0.16^b^	2.00 ± 0.16^b^	p = 0.85
Isoleucine	0.54 ± 0.06^a^	0.62 ± 0.07^a,b^	0.71 ± 0.07^b^	p = 0.89
Leucine	1.31 ± 0.19^a^	1.69 ± 0.20^a^	2.86 ± 0.20^b^	p = 0.21
Lysine	2.72 ± 0.27^a^	1.40 ± 0.29^b^	1.28 ± 0.29^b^	p = 0.54
Methionine	0.27 ± 0.03^a^	0.35 ± 0.04^a^	0.48 ± 0.04^b^	p = 0.17
Phenylalanine	0.51 ± 0.08^a^	0.89 ± 0.08^b^	1.04 ± 0.08^b^	p = 0.69
Threonine	4.77 ± 0.49^a^	6.11 ± 0.53^b^	7.33 ± 0.53^b^	p = 0.08
Tryptophan	nd^4^	nd	nd	---
Valine	2.36 ± 0.23^a^	3.76 ± 0.25^b^	4.57 ± 0.25^c^	p = 0.44
**Non-essential FAAs:**				
Alanine	12.54 ± 1.56^a^	21.33 ± 1.69^b^	27.27 ± 1.69^c^	p = 0.46
Arginine	1.56 ± 0.18^a^	1.05 ± 0.19^b^	0.95 ± 0.19^b^	p = 0.87
Aspartic acid	2.41 ± 0.48^a^	3.78 ± 0.52^b^	5.56 ± 0.52^c^	p = 0.31
Asparagine	nd	nd	nd	---
Cysteine	0.65 ± 0.15^a^	1.28 ± 0.16^b^	2.24 ± 0.16^c^	p = 0.58
Glutamic acid	44.44 ± 5.96^a^	91.25 ± 6.45^b^	120.33 ± 6.45^c^	p = 0.19
Glutamine	10.70 ± 2.64^a^	33.40 ± 2.86^b^	61.15 ± 2.86^c^	p = 0.48
Glycine	4.94 ± 0.73^a^	9.74 ± 0.78^b^	13.23 ± 0.78^c^	p = 0.76
Proline	3.70 ± 0.35^a^	2.68 ± 0.38^b^	2.61 ± 0.38^b^	p = 0.89
Serine	4.12 ± 0.61^a^	8.43 ± 0.66^b^	12.55 ± 0.66^c^	p = 0.69
Taurine	51.24 ± 2.39^a^	42.49 ± 2.58^b^	35.72 ± 2.58^c^	p = 1.00
Tyrosine	0.68 ± 0.09^a^	0.95 ± 0.10^b^	1.02 ± 0.10^b^	p = 0.43
**Totals**				
Essential	13.93 ± 1.09^a^	17.14 ± 1.17^b^	20.27 ± 1.17^c^	p = 0.13
Non-essential	136.98 ± 11.35^a^	216.31 ± 12.26^b^	282.54 ± 12.26^c^	p = 0.67
All FAAs	150.91 ± 12.00^a^	233.44 ± 12.97^b^	302.82 ± 12.97^c^	p = 0.79

^1^Data are expressed as Least Squared Means (LSM) ± SEM. Estimates created from Mixed effects model.

^2^Classification of FAA based on Dietary Reference Intakes^13^.

^3^Different letters are statistically different from each other.

^4^nd = not detectable; below the limit of detection.

## Discussion

The FAA profile in breast milk changed during the first four months of lactation: most FAAs increased as lactation progressed, whereas lysine, arginine, taurine and proline decreased with time. Tryptophan and asparagine were undetectable at all time points; most likely due to use of UV/VIS spectrometry in the detection of amino acid-ninhydrin derivatives. Maternal age did not have a significant linear relationship with any of the FAAs. Of all the FAAs, glutamic acid, glutamine, taurine and alanine were abundant with the two former FAA accounting for about 50% of total FAAs. These findings are relatively consistent with prior research in human (Rassin et al. 1978; Harzer et al. 1984; Agostoni et al. 2000a) and porcine (Wu et al. 2011) milk.

The abundance of FAAs in human milk seems to be a constant trait across age (Armstrong and Yates 1963; Ghadimi and Pecora 1963; Svanberg et al. 1977). However, in the present study many FAA levels were lower than previously reported (Agostoni et al. 2000b). We suggest two explanations, not mutually exclusive. First, although all mothers were primiparous, of normal or overweight status, delivered healthy term infants vaginally, and successfully breastfed for several months, many were Andean Mestizo women whose socio-economic status was low. Further, although common for adolescent births, many of the babies had weight-for-age z-scores <0 at study entry. Whether dietary habits and nutritional status of the mothers (Sanchez-Llaguno et al. 2013) may have been a contributing factor remains unknown.

Second, aspects of the experimental protocol may have resulted in lower FAA levels. In the present study milk samples were collected after an overnight fast. While the levels of free glutamate in the milk of lactating women collected after 8 h overnight fasting (Stegink et al. 1972) are comparable to the ones found in the present study, levels of FAA in milk appear to change throughout the day (Sánchez et al. 2013). Nevertheless, future studies should be conducted that compare levels of FAA in milk under experimentally controlled fasting and fed conditions. Because animal studies suggest that dietary branched-chain amino acids (BCAA) contribute to the synthesis of free and protein-bound glutamate, glutamine, aspartate and asparagine in milk (Li et al. 2009; Lei et al. 2012), the short-term regulation of FAA content and the effects of dietary history in the short- and long- term in humans are important areas for future research.

The content of FAAs represents a small fraction (3–5%) of total amino acids in human milk (Svanberg et al. 1977; Agostoni et al. 2000b). However, this is 100 times higher than the 0.05% FAA pool in tissues and even higher than the FAA pool in plasma (Christensen 1964). We found that the concentration of most FAAs is higher in mature milk than in colostrum or transitional milk. Because the total protein content of human milk decreases rapidly during the first month of lactation (Lonnerdal et al. 1976), as the baby grows, human milk contains more FAAs relative to total protein. What remains a mystery is the specific role that FAAs play in infant development and why the mammary tissue of humans and other mammals have develop a mechanism to deliver FAAs and specially glutamate and glutamine through milk? Because of its unbound nature, FAAs can be sensed by receptors in both the oral cavity (Chaudhari et al. 2000) and intestinal and gastric walls (San Gabriel and Uneyama 2013), likely conferring beneficial physiologic effects (e.g., gut development (Burrin et al. 2008) and satiety (Mennella et al. 2011; Ventura et al. 2012).

In summary FAA concentrations in human breast milk change over the first four months of lactation but are not related to mother’s age. However, more research is needed to further explore the role of maternal age and nutritional status on the quality of her milk. Understanding the nature of these changes will help us develop better nutritional support for mothers and infants around the world.
